# Comparative genomics of whole-cell *pertussis* vaccine strains from India

**DOI:** 10.1186/s12864-020-6724-8

**Published:** 2020-05-07

**Authors:** Shweta Alai, Vikas C. Ghattargi, Manish Gautam, Krunal Patel, Shrikant P. Pawar, Dhiraj P. Dhotre, Umesh Shaligram, Sunil Gairola

**Affiliations:** 1 Department of Health and Biological Sciences, Symbiosis International University, Pune, Maharashtra 412115 India; 2 National Centre for Microbial Resource, National Centre for Cell Science, Pune, Maharashtra 411021 India; 3 Serum Institute of India Pvt. Ltd, Pune, Maharashtra 411028 India

**Keywords:** *Bordetella pertussis*, Whooping cough, Resurgence, Antigenic variation, Genome organization, Virulence genes, Vaccine-mediated selection

## Abstract

**Background:**

Despite high vaccination coverage using acellular (ACV) and whole-cell pertussis (WCV) vaccines, the resurgence of pertussis is observed globally. Genetic divergence in circulating strains of *Bordetella pertussis* has been reported as one of the contributing factors for the resurgence of the disease. Our current knowledge of *B. pertussis* genetic evolution in circulating strains is mostly based on studies conducted in countries using ACVs targeting only a few antigens used in the production of ACVs. To better understand the adaptation to vaccine-induced selection pressure, it will be essential to study *B. pertussis* populations in developing countries which are using WCVs. India is a significant user and global supplier of WCVs. We report here comparative genome analyses of vaccine and clinical isolates reported from India. Whole-genome sequences obtained from vaccine strains: WCV (J445, J446, J447 and J448), ACV (BP165) were compared with Tohama-I reference strain and recently reported clinical isolates from India (BPD1, BPD2). Core genome-based phylogenetic analysis was also performed using 166 isolates reported from countries using ACV.

**Results:**

Whole-genome analysis of vaccine and clinical isolates reported from India revealed high genetic similarity and conserved genome among strains. Phylogenetic analysis showed that clinical and vaccine strains share genetic closeness with reference strain Tohama-I. The allelic profile of vaccine strains (J445:*ptxP1/ptxA2/prn1/fim2–1/fim3–1*; J446: *ptxP2/ptxA4/prn7/fim2–2/fim3–1*; J447 and J448: *ptxP1/ptxA1/ prn1/fim2–1/fim3–1*), which matched entirely with clinical isolates (BPD1:*ptxP1/ptxA1/prn1/fim2–1* and BPD2: *ptxP1/ptxA1/prn1/fim2–1*) reported from India. Multi-locus sequence typing (MLST) demonstrated the presence of dominant sequence types ST2 and primitive ST1 in vaccine strains which will allow better coverage against circulating strains of *B. pertussis*.

**Conclusions:**

The study provides a detailed characterization of vaccine and clinical strains reported from India, which will further facilitate epidemiological studies on genetic shifts in countries which are using WCVs in their immunization programs.

## Background

Whooping cough (Pertussis) is a respiratory disease caused by the Gram-negative bacterium *Bordetella pertussis* [1]. The introduction of whole-cell vaccines (WCVs) in the 1950s and switch to acellular pertussis vaccines (ACVs) targeting a few virulent proteins in the 1990s played a central role in the control of whooping cough [2–5]. In the last decade, despite high vaccination coverage, pertussis has unexpectedly reemerged in several countries [6–12]. Several possible hypotheses were proposed for the resurgence, such as waning of vaccine-induced immunity, improved surveillance and diagnosis of the disease, and genetic divergence among the strains [13–15].

Genetic divergence was mostly studied in circulating strains of *B. pertussis* concerning vaccine antigens such as pertussis toxin (ptx), pertactin (prn), fimbriae (fim) and filamentous hemagglutinin (FHA) [16–19]. The pathogen adaptation in clinical strains was also observed with respect to the emergence of antigen deficient strains [20]. The circulating strains deficient for prn, FHA, and ptx were reported in several countries [21–23]. Pertactin deficient strains were first reported in Philadelphia, USA and later found in many countries like France, Japan, Australia, Finland and Italy, where ACVs have been used [24, 25]. There are also views that adaptation of pertussis strains goes beyond the changes in ACVs associated proteins and involves other virulence-associated factors and surface-exposed proteins [26]. Strains deficient in tracheal colonization factor (virulence associated protein in *B. pertussis*), were reported from Belgium, Netherland, and the USA [27]. Besides antigenic divergence, massive gene loss, pseudogenes formation and insertion sequence (IS481) mediated genomic rearrangements are among the significant genomic features of the *B. pertussis* adaptation that became apparent in different comparative genomic studies [28–35]. Recently, a comparative genomic study based on 343 *B. pertussis* isolates primarily from countries using ACV suggested that adaptive evolution of this pathogen is closely associated with vaccine introduction and emergent strains spread rapidly between countries [28].

Conventional approaches used to study the population of *B. pertussis* include serotyping, genotyping for the key protective antigens and pulsed-field gel electrophoresis (PFGE) [36–38]. While high throughput, these approaches are limited by their sensitivity to detect minor genetic variations within the genome. Whole-genome sequencing of *B. pertussis* isolates and vaccine strains are better suited to understand the impact of vaccination strategies on pathogen diversity. Our current knowledge of *B. pertussis* adaptation is based on studies in countries that are using ACVs. WCVs are based on the use of inactivated whole-cell as an antigen and therefore induce a broader immune response. Thus, to develop effective strategies to prevent pertussis, it is crucial to study *B. pertussis* adaptation globally including countries which are using WCV [35].

WCVs are commonly used in developing countries, and among them, India is the largest global supplier of WCVs and is primarily using WCVs in their immunization program [39–41]. We reported whole genome sequences of WCV and ACV strains of *B. pertussis* from India [42, 43]. Genome sequences of two Indian clinical isolates BPD1 (CP034102) and BPD2 (CP034101) are reported. These are the only two isolates reported from India to date [44].

We report here comparative genomic analysis of five vaccine strain and two clinical isolates from India with the reference strain. Phylogenetic analysis of these vaccine strains and isolates was also performed using 166 isolates reported from countries which are using ACV. Such data will provide opportunities for facilitating surveillance of pertussis in India and its comparisons with globally reported trends in *B. pertussis* populations.

## Results

### General genome features

Comparison of general genomic characteristics of five vaccine strains (J445, J446, J447, J448, BP165), and two clinical (BPD1, BPD2) isolates with Tohama-I are summarized in Table 1. Tohama-I have been employed as a reference strain in the study as its genome is completely sequenced and well characterized. Additionally, Tohama-I has been used as a reference in most comparative genomic studies [33, 38]. The average genome size reported for *B. pertussis* strains is 4.1Mbp, strains J447 and J448 reported slightly higher size of 4.2Mbp and 4.3Mbp respectively. Percent G + C for all strains was observed in the range of 67.12 to 67.82%, and gene encoding regions (CDS) were in the range of 3876 to 4128, which is consistent with the reported values for *B. Pertussis* strains (Table 1). *B. pertussis* strains were reported to have genomic deletions and intra-genomic rearrangements through IS copy number expansion, predominantly for IS481 (~ 250 copies) [30, 33, 38, 46, 47]. The *B. pertussis* genome is reported to have ~ 238 copies of IS481, ~ 17 copies of IS1663 and ~ 6 copies of IS1002. The clinical and vaccine strains copy numbers of IS481, IS1663 and IS1002 were found to be comparable to reported *B. pertussis* genomes.

**Table 1:** General genome features of strains

Feature	Vaccine Strains					Clinical Isolates		Reference Strain
Vaccine	WCV	WCV	WCV	WCV	ACV			
Strains	J445	J446	J447	J448	BP 165	BPD1	BPD2	Tohama –I
Size (bp)	4,128,984	4,140,370	4,257,407	4,386,396	4,101,762	4,126,211	4,104,911	4,086,189
G + C Content	67.72	67.71	67.77	67.82	67.71	67.12	67.14	68.12
Genes	3940	3951	4069	4192	3893	4004	3985	3856
Coding sequences	3876	3887	4005	4128	4035	3941	3921	3806
Pseudo-genes	231	237	238	246	307	384	359	359
rRNA (5S, 16S, 23S)	3, 3, 3	3, 3, 3	3, 3, 3	3, 3, 3	3,3,3	3,3,3	3,3,3	3,3,3
tRNA	51	51	51	51	49	51	51	51
IS481	255	252	266	273	253	248	251	238
IS1663	19	19	21	23	18	20	19	17
IS1002	8	5	8	8	8	7	7	6
Accession number	CP017402	CP017403	CP017404	CP017405	RSFF00000000	CP034182	CP034101	NC002929
Sequencing Platform	PacBio RSII, Illumina MiSeq	PacBio RSII, Illumina MiSeq	PacBio RSII, Illumina MiSeq	PacBio RSII, Illumina MiSeq	Illumina MiSeq	Ion- Torrent Oxford NanoporeMiniON	Ion- Torrent Oxford NanoporeMiniON	Illumina NextSeq
Contigs	Complete	Complete	Complete	Complete	264	Complete	Complete	Complete
N50	Complete	Complete	Complete	Complete	68,043	Complete	Complete	Complete
Coverage (x)	144	226	239	229	100	203	195	–
Reference	42	42	42	42	42	44	44	33

The insertion of IS elements is known to create pseudogenes in *B. pertussis* genomes [3, 38]. The pseudogenes were also studied in vaccine and clinical isolates as compared to Tohama-I (Table 1). The number of pseudogenes in vaccine strains ranged between 231 to 307, which were lower than Tohama-I. Whereas, clinical isolates pseudogenes ranged from 359 to 384 and were comparable to Tohama-I which reported 359 pseudogenes.

Vaccine and clinical strains genomic similarity (symmetric identity) was assessed using NCBI genome neighbor report (Additional file 1). Vaccine strains and clinical isolates displayed more than 95% similarity with Tohama-I (Table 2)

**Table 2:** Symmetric identity (Genome similarity) between strains based on NCBI genome neighbor report

Comparator	Vaccine Strains				Clinical Isolates	
	J445	J446	J447	J448	BPD1	BPD2
**Tohama-I**	98.7038	98.6752	97.1793	95.6999	97.8997	98.5198
**J445**	–	98.908	98.3484	96.8588	98.9038	99.69
**J446**	98.908	–	97.3909	95.9258	98.0163	98.7242
**J447**	98.3484	97.3909	–	98.5076	97.4165	98.0679
**J448**	96.8588	95.9258	98.5076	–	95.9406	96.5784
**BPD1**	98.9038	98.0163	97.4165	95.9406	–	99.1836
**BPD2**	99.69	98.7242	98.0679	96.5784	99.1836	–

Column 1 in table represents the comparator against which the strains (highlighted in rows) were compared

### Pan-genome analysis

**Fig. 1:**
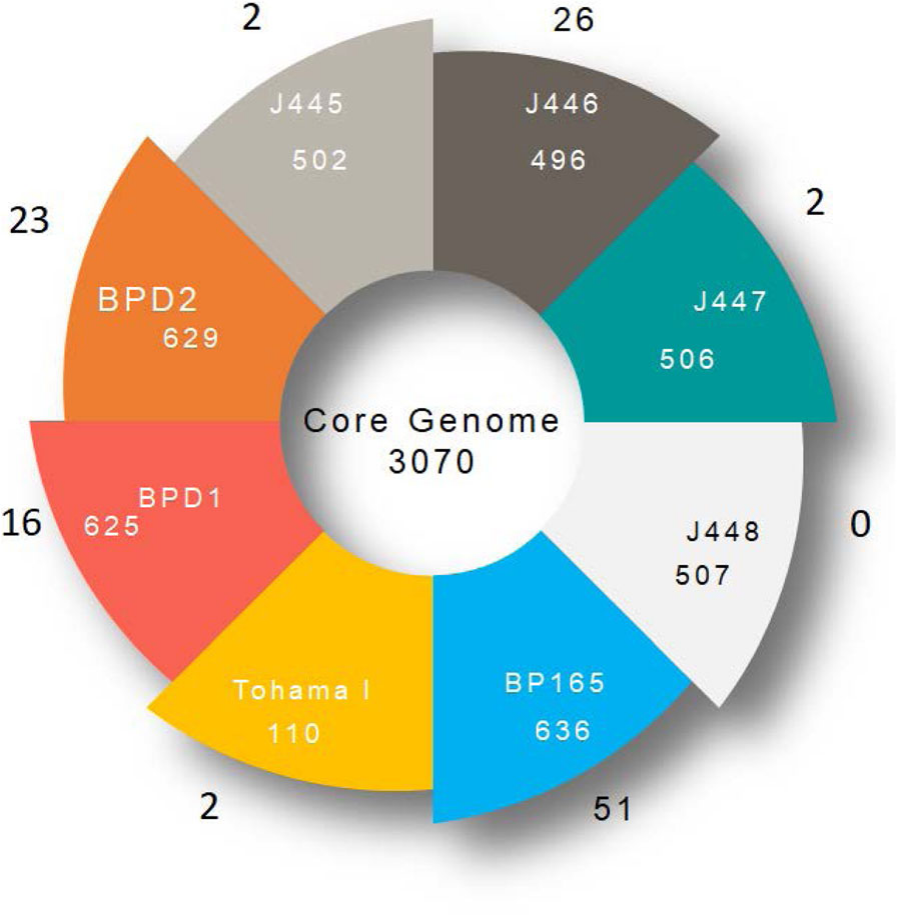
Circular genome representation of vaccine and clinical strains with reference strain. Circular diagram of pan-genome of vaccine strains (J445, J4445, J446, J448, BP165) and clinical strains (BPD1, BPD2) and reference strain Tohama-I. The intersection of all strains presents the total number of core genomes. The intersection of each pair represents the total number accessory genome for all strains, while the outer number represents the total number of unique genes associated with the strains

**Fig. 2:**
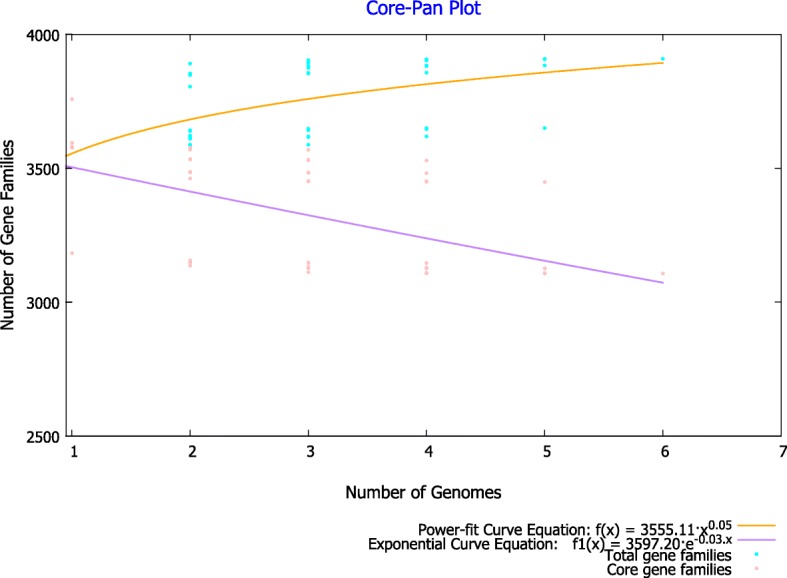
Pan and core genome plot. Pan Genome and core genome plot of eight *B. pertussis* vaccine strains (J445, J4445, J446, J448, BP165) and clinical strains (BPD1, BPD2) and reference strain Tohama-I. The plot shows that progression of the pan (orange) and core (purple) genomes. The number of shared genes was plotted as the function of the number of strains (n) added sequentially with 3070 genes which were shared by genomes. The orange line represents the least-squares fit to the power-law function f(x) = a.x^b where a = 3538.18, b = 0.0289134. The red line represents the least-squares fit to the exponential decay function f1(x) = c.e^ (d.x) where c = 3672.32, d = − 0.0317036

The pan-genome of 5 vaccines, 2 clinical and 1 reference strain was made up of 3980 genes (Fig. 1). This size is comparable with the pan-genome of 171 *B. pertussis* strains collected mostly from ACV using countries, which consisted of 3871 genes [49]. The core genome of eight strains consisted of 3070 genes, which constitute approximately 77% CDSs of these strains. Such high percentage of core genes suggests a low level of genomic diversity among vaccine and clinical strains [50]. The pan-genome curve was generated by plotting the total number of distinct gene families against the number of genomes used in this study (Fig. 2). Similarly, the number of shared gene families was plotted against the number of genomes to generate the core-genome plot. BPGA calculates the pan-genome size and core genome size for the given “N” genomes [49]. The power-law regression model and an exponential curve fit model were calculated for all strains used in this study. Power law regression model suggested as “open but slowly closing pan-genomes”. The pan-genome model calculated as y = a.bx^c^ (where a,b,c is parameters) (Fig. 2). Pan-genome size (n) with sequenced genomes (N), was modelled as n = kNɤ, where open pan-genome has ɤ value greater than zero and less than one. These lower values signifying a more closed genome with fewer acquired genes. The ɤ value for the classical *Bordetella* subspecies (0.090) which was lower than that of *Bacillus cereus* (0.43), indicating the pan-genome is open but slowly closing [50–52]. Previous studies predicted that *B. anthracis* has a closed pan-genome based only on five available genomes (α = 5.6 > 1) [51]. This preliminary data suggests that the pan-genome of strains appears open but slowly closing.

**Fig. 3:**
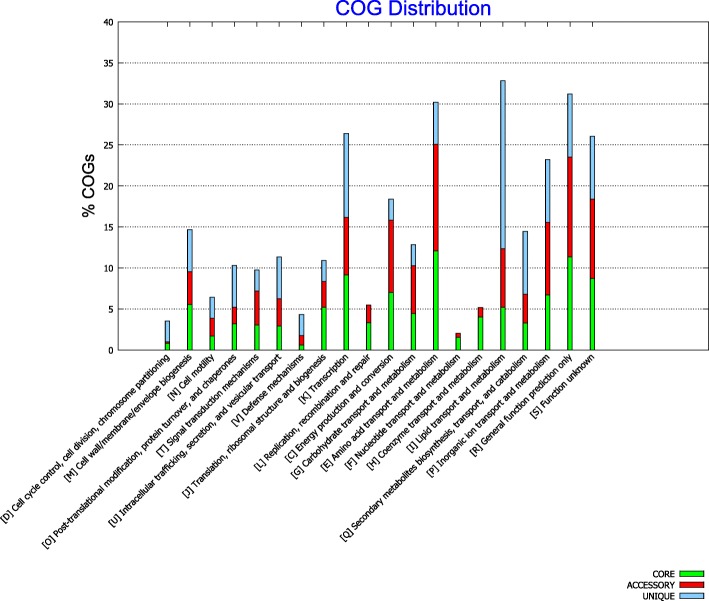
Functional annotation with Clusters of Orthologous Genes (COGs). Functional annotation with Clusters of Orthologous Genes (COGs) assigned pan-genome of vaccine strains (J445, J4445, J446, J448, BP165) and clinical strains (BPD1, BPD2) and reference strain Tohama-I. The height of each bar represents a percentage of the core, accessory and unique genes involved in specific functional categories represented at the horizontal axis

To estimate a general functional role of the CDS present in the average genome of eight strains used in this study, the clusters of orthologous groups of proteins (COGs) for each of the genome were determined. The top four COG categories observed in all eight genomes were designated as, I (Lipid transport and metabolism, E (Amino acid transport and metabolism), K (Transcription) and P (Inorganic ion transport) while the lowest category containing COG genes were F (Nucleotide transport and metabolism), D (Cell cycle control, cell division, chromosome partitioning) and N (cell motility) (Fig. 3). A total 23 functional categories were defined in the Tohama-I strain according to COG analysis [32]. In comparison with the reference strain, only 20 functional categories were observed for eight strains used in this study. The categories absent in vaccine and clinical genomes as compared to Tohama-I were related to genes involved in nucleotide metabolism, membrane transport and iron metabolism.

### Mobilome analysis

Mobilome or mobile genetic elements (MGEs) include insertion sequences, bacteriophages, and genomic islands (GIs). *B. pertussis* genome has more than 200 copies of insertion sequence (IS elements) [33, 34]. ISs present in 5 vaccine and 2 clinical strains mainly belonged to three IS families IS481, IS1002, IS1663 (Additional file 2). The average copy number observed for IS481, IS1001 and IS1002 of genomes used in this study was 257, 20 and 7, respectively as discussed earlier (Table 1). We observed a slight increase in the copy number of IS481 similar with reports from countries using ACV.

PHASTER tool was used to identify phage region in all genomes in this study. Potential prophage sequences in the genome were identified and categorized as intact, incomplete or questionable [59]. Only in clinical isolate BPD2, we observed one intact phage region called as phage 1 which consists of 20.3 kb (1627048–1,647,419 bp) region having 62.30% G + C content and a total of 24 CDS. Phage 1 region from BPD2 were typically found to contain several phage-associated genes (Additional file 2). Clinical isolate BPD1 also showed the presence of 5 regions, but none was intact (4 incomplete and 1 uncharacterized region) (Additional file 2). Vaccine strain J445, J446, J448 and other *B. pertussis* strains carried phage region similar to phage 1. We also observed the presence of a similar region in other *B. pertussis* complete genomes available in databases by using BLAST analysis. Approximately 18% (100 out of 551) of available complete *B. pertussis* genomes showed high similarity with phage 1. We did not observe CRISPR sequences, plasmids and antibiotic resistance genes in any of the genomes.

**Fig. 4:**
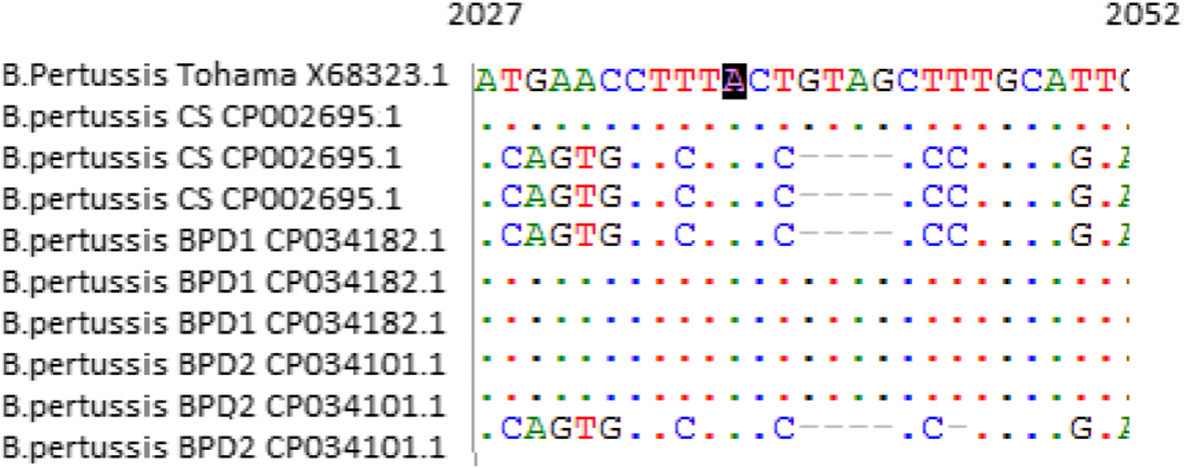
Identification of single nucleotide polymorphism associated with antibiotic resistance using multiple genome alignment. Multiple sequence alignment generated using MEGA 9, with sequence of the 23S rRNA gene of *Bordetella pertussis as* reference sequence, showing sequence similarity of clinical strains BPD1 and BPD2 from India with 23S rRNA gene of *Bordetella pertussis* of Tohama-I and Chinese vaccine strain at position A2047G based on old Tohama-I 23S rRNA gene of Tohama-I. Absence of nucleotide variation observed at position describing mutation associated with antibiotic resistance (binding site of erythromycin)

The mutation associated with antibiotic (macrolide) resistance observed were from A to G at position 2047 (A2047G) located in domain V of the 23S rRNA gene [63]. The reported A2047G position was based on old Tohama-I 23S rRNA sequence, and it is equivalent to A2037G in the updated Tohama-I genome (Accession No. NC_002929.2). The 23S rRNA gene sequence of clinical isolates BPD1 and BPD2 were compared with 23S rRNA gene Tohama-I (X68323) and Chinese vaccine strain (CP002695), as all the PCR based diagnostic tools to detect the antimicrobial resistance mechanism in *B. pertussis* strains were based on these reference genomes. Based on whole genome sequence data we did not observe such mutation in clinical isolates BPD1 and BPD2 reported from India (Fig. 4).

Genomic plasticity reported in *B. pertussis* is through gene acquisition, gene loss and genomic organization [64]. Horizontal gene transfer (HGT) is also one of the mechanisms responsible for genome evolution. Genomic islands (GIs) are genomic fragments acquired by HGT events and may have an impact on the genome plasticity. We observed 31 GIs in BPD1 and BPD2 strains consisting of 484 and 528 genes, respectively (Additional file 3). Most of these genes were involved in carbohydrate, amino acid metabolism, membrane transport and transposases.

### cgMLST and phylogenetic analysis

Genome sequences of vaccine and clinical strains were analyzed using the gene-by-gene approach known as core genome MLST (cgMLST). Recently cgMLST genotyping strategies were implemented for international coordinated surveillance of several pathogenic bacterial species. cgMLST has been recently developed for *B. pertussis* surveillance [45]. cgMLST scheme provides an excellent approach that combines high resolution of genome-level variation with high reproducibility. We compared vaccine and clinical strain genome sequences with the *Bordetella spp*. database (https://bigsdb.pasteur.fr/bordetella/). We recorded individual strain matching profiles, cgST profiles and number of mismatches with predefined 2038 core gene loci for each genome. Global comparison of Indian clinical *B. pertussis* isolates BPD1 and BPD2 with cgMLST database revealed 97.2% (1981/2038) and 94.9% (1935/2013) similarity, respectively. Among the 2038 loci of the cgMLST scheme, 57 (BPD1) and 103 (BPD2) loci showed differences with cgMLST database (Additional file 4). The cgST profiles were found to be similar for both clinical isolates (Table 3).

**Table 3:** MLST and cgMLST profiles of strains

Feature	Strain	adk	FumC	glyA	TyrB	Icd	pepA	Pgm	ST	cg-MLST
Vaccine strains	J445	1	1	1	3	1	1	1	2	413
	J446	1	1	1	1	1	1	1	1	410
	J447	1	1	1	3	1	1	1	2	411
	J448	1	1	1	3	1	1	1	2	412
	BP 165	1	1	1	3	1	1	1	2	41
Clinical isolates	BPD1	1	1	1	3	1	1	1	2	362
	BPD2	1	1	1	3	1	1	1	2	362

**Fig. 5:**
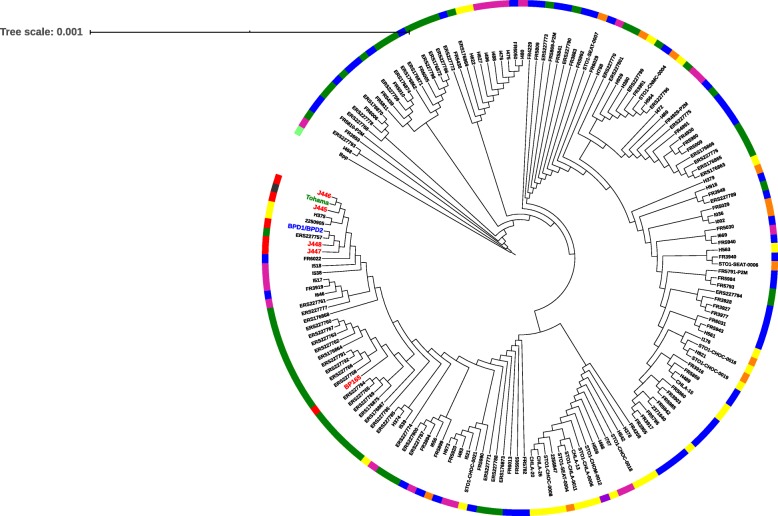
Phylogenetic analysis of vaccine and clinical strains using cgMLST. Maximum-likelihood phylogenetic tree for Indian *Bordetella pertussis* isolates and vaccine strains and isolates from other geographical origins based on concatenated alignments of 2038 cgMLST gene sequences. The tree was rooted with strain *Bordetella parapertussis* (GenBank Accession number CP019931.1). Tree scale is indicated above the diagram. The external circle indicates the geographical origin of isolates (blue, France; red, India; Pink, Vermont; Green, UK; Yellow, California; Orange, Washington; Purple, Michigan). Vaccine strains and Indian Isolates labels highlighted in bold red colour. The scale bar indicated amino-acid substitution per site. The tree was constructed with bootstrap values 1000 replicates. Bootstrap values are indicated with the position on branch 50 and scale by factor 1

Phylogenetic analysis of 5 vaccines, 2 clinical isolates reported from India and reference strain was compared with 166 isolates from countries using ACVs. These isolates represent regions corresponding to France, US and UK [45]. Of these 166 isolates, 55 isolates from France corresponded to groups of intrafamilial or of multiple isolates from the same patient and randomly selected cocirculating isolates. Out of the remaining 111 isolates, corresponding to 3 outbreaks of pertussis that observed in ACV using countries like US and UK [57]. Core genome phylogenetic tree was constructed using amino acid sequences from cgMLST loci extracted for 166 *B. pertussis* isolates [45]*.* Phylogenetic tree was constructed with *Bordetella parapertussis* as an outgroup (Fig. 5). Phylogenetic analysis using 2038 core gene sequences of four Indian *B. pertussis* vaccine strains (J445, J446, J447 and J448) showed close genetic relatedness with Indian clinical isolates and Tohama-I (bootstrap 80). Vaccine strain J445, J446 formed a separate sub-cluster with Tohama-I (bootstrap 99) and strain J447 and J448 shared separate sub-cluster with isolates BPD1, BPD2 (Fig. 5). Interestingly, isolates H3755, 2,250,905, ERS227757 and FR6022 were found to be closely related with Indian vaccine strains and clinical isolates (bootstrap 80). Isolates H3755, 2,250,905 from US (California), ERS227757 from UK and FR6022 from France also shared closeness with Tohama-I. The closeness was further consistent with allelic profiles as isolates showed similar genetic profiles as *ptxP1, ptxA1, fim2–1, fim3–1* with Tohama-I [45]. Out of all the vaccine strains, BP165 was found to be distant from Indian clinical isolates. This could be attributed to the origin of BP 165, as it is a US isolate. BP165 clustered closely with isolates ERS227758 reported from UK with a similar *fim2–1, prn1* allele profile. BP165 was also found to form a separate sub-cluster with isolate ERS227764 having *PtxP3* allele.

### MLST and genotyping

*Bordetella* MLST database classifies *Bordetella* genus into 43 sequence types (STs) and 4 clonal complexes (CCs). Of these CCs, CC2 belongs to *B. pertussis* and is composed of 3 sequence types, ST1, ST2, and ST24 [1, 65]. Sequence type (ST-2) reportedly covers most of the circulating strains and is a dominant sequence type since late 1990s [65]. ST1 profile represents largely ancient strains such as Tohama-I [61, 62]. MLST analysis suggests that four vaccine strains (J445, J447, J448 and BP165) and two clinical isolates (BPD1 and BPD2) belong to ST-2 class (Table 3). Whereas, J446 strain showed ST-1 profile.

Globally, *B. pertussis* isolates are characterized based on allelic profiles of major virulence genes including promoter sequence of pertussis toxin gene (ptxP), pertussis toxin (ptxA), pertactin (prn) and fimbriae (fim2 and fim3) [66, 67]. These virulence-associated genes have shown divergence from vaccine reference strains [16–19]. Vaccine and clinical isolates were subjected to allelic profiling. Both Indian clinical isolates showed allele profiles as (BPD1:*ptxP1/ptxA1/prn1/fim2–1* and BPD2: *ptxP1/ptxA1/prn1/fim2–1*) which were found similar to WCV vaccine strains (J445:*ptxP1/ptxA2/prn1/fim2–1/fim3–1*; J446: *ptxP2/ptxA4/prn7/fim2–2/fim3–1*; J447 and J448: *ptxP1/ptxA1/ prn1/fim2–1/fim3–1*). We also studied allelic profiles of other virulence-associated genes such as tracheal colonization factor (tcf-A), *Bordetella* associated protein C (BapC), Adenylate cyclase (cyaA), outer membrane protein Q and Virulence associated gene (Vag8) in vaccine and clinical isolates. The Indian clinical isolates and vaccine strains demonstrated similar profiles as (*tcfA-2-tcfA-9, bapC1, cyaA2, ompQ1-ompQ2, vag8*).

### Gene loss and duplication

Number of genes lost or duplicated in clinical and vaccine strains was studied as compared to reference strain Tohama-I. The study suggests that vaccine strains and clinical isolates displayed gene loss and duplication with no significant impact on overall genome size as the number of gene lost was nearly equal to the number of genes duplicated (Table 4). Genomic deletions and ongoing gene loss are one of the apparent features observed in recent clinical isolates reported from many countries [44, 55]. The lost genes majorly belonged to categories of membrane transport and amino acid metabolism. The genes identified in the category of amino acid metabolism were related to ABC transporter’s components. ABC transporters are a large group of proteins which have cellular functions in import and export various substances, which suggests deletion of such genes might confer a selective advantage as a one of the strategies during adaptation of the organism in highly vaccinated populations [44, 48, 68]. The duplicated genes in all strains found in COG categories belonged to amino acid and nucleic acid metabolism and post-translation modification (Additional file 5). We did not observe any gene loss or gene acquisition of virulence-associated genes.

**Table 4:** Gene loss and duplication in strains

Feature	Strains	No of absent genes	No of gain genes	No of duplicated genes	No of genes defined in COG (%)
Vaccine Strains	J445	393	36	388	–
	J446	393	37	387	18
	J447	393	36	448	–
	J448	394	36	446	0
Clinical strains	BPD1	401	36	401	–
	BPD2	395	36	388	–
Reference strain	Tohama-I				387

### Whole genomic comparison

**Fig. 6:**
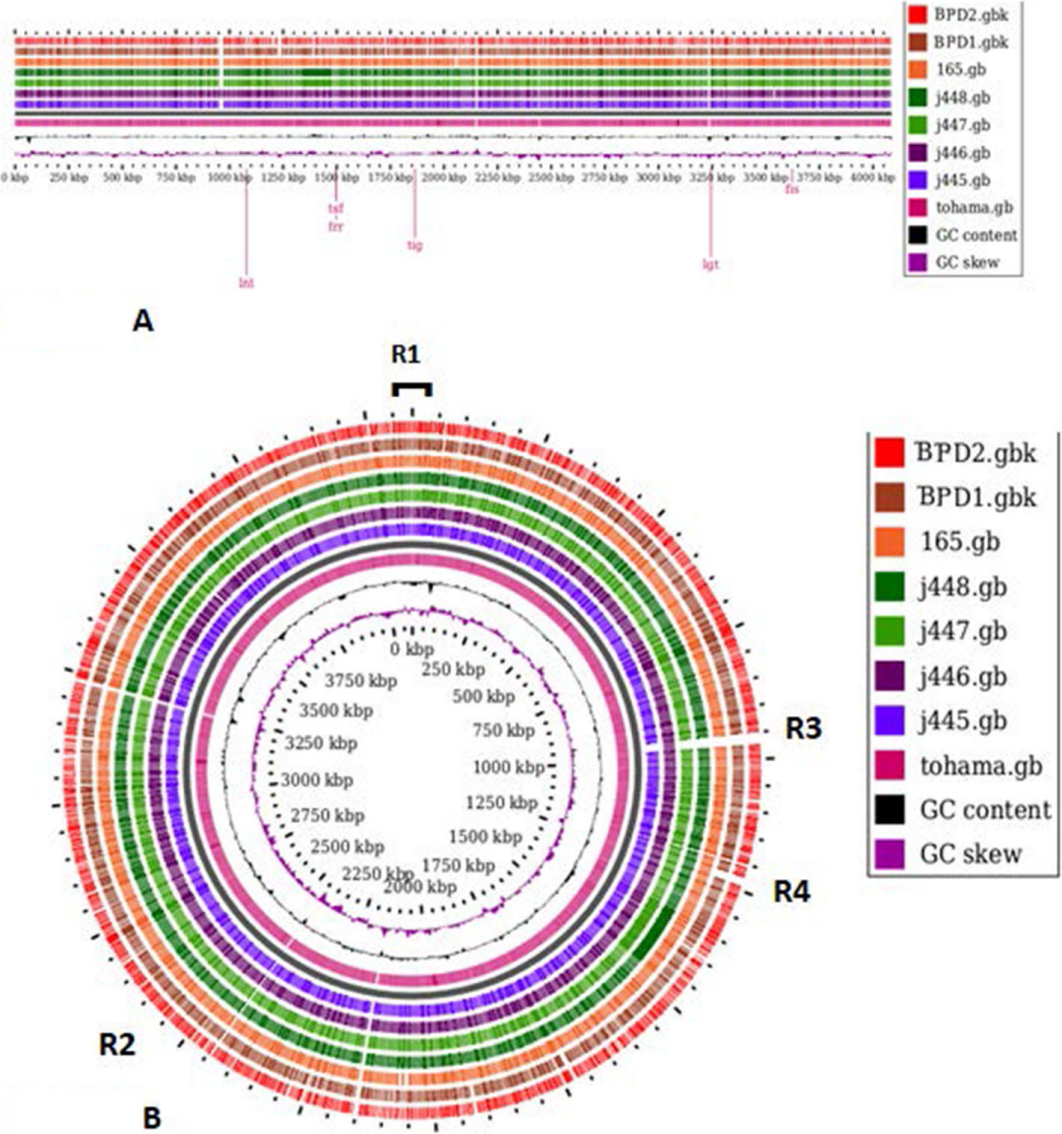
BLAST Atlas comparing eight *B. pertussis* strains. BLAST Atlas comparing eight *B. pertussis* vaccine strains (J445, J4445, J446, J448, BP165) and clinical strains (BPD1, BPD2) against reference strain Tohama-I. a. The linear arrangement of genomes of the vaccine strain, clinical strain genomes similarity with reference strain Tohama-I. b. Circular plot generated using G view Comparison Tool using BLASTn. Genomes are arranged with vaccine strains and clinical strains from innermost ring close to the reference genome to outermost ring from reference genome: inner circle reference strain Tohama-I (pink) GC content (black), G + C skew (purple)

A BLAST Atlas comparing all seven genomes with reference genome was generated using the Gview comparison tool with e value 1e-10 and percent identity cutoff (removing matches below percent identity) of 80%. A circular genome map with each genome represented by the ring was constructed with center circle representing Tohama-I. A visual inspection of the circular alignment of the genomes revealed a high sequence similarity in all genomes with reference genome; especially in the region 1 (approximately1–850 bp and the region of 3250–3780 bp) (Fig. 6). This region was identical in all strains with 100% identity (Additional file 6). Also, region 2 (1500–3000 bp) showed more than 99% identity. We found a small gap (region 3) in strains J445, J447, J448, BPD1 and BPD2 between 900 and 1000 bp (Fig. 6). The genes observed in this gap were related to amino acid and nucleotide metabolism. We also observed a small gap in region 4 (1300-1500 bp) in clinical strains as compared to vaccine strains. The circular genome map comparison showed that all strains especially clinical strains are identical with vaccine and reference strain, with slight gene loss detected. All the absent genes in clinical isolates were involved in amino and nucleotide metabolism.

**Fig. 7:**
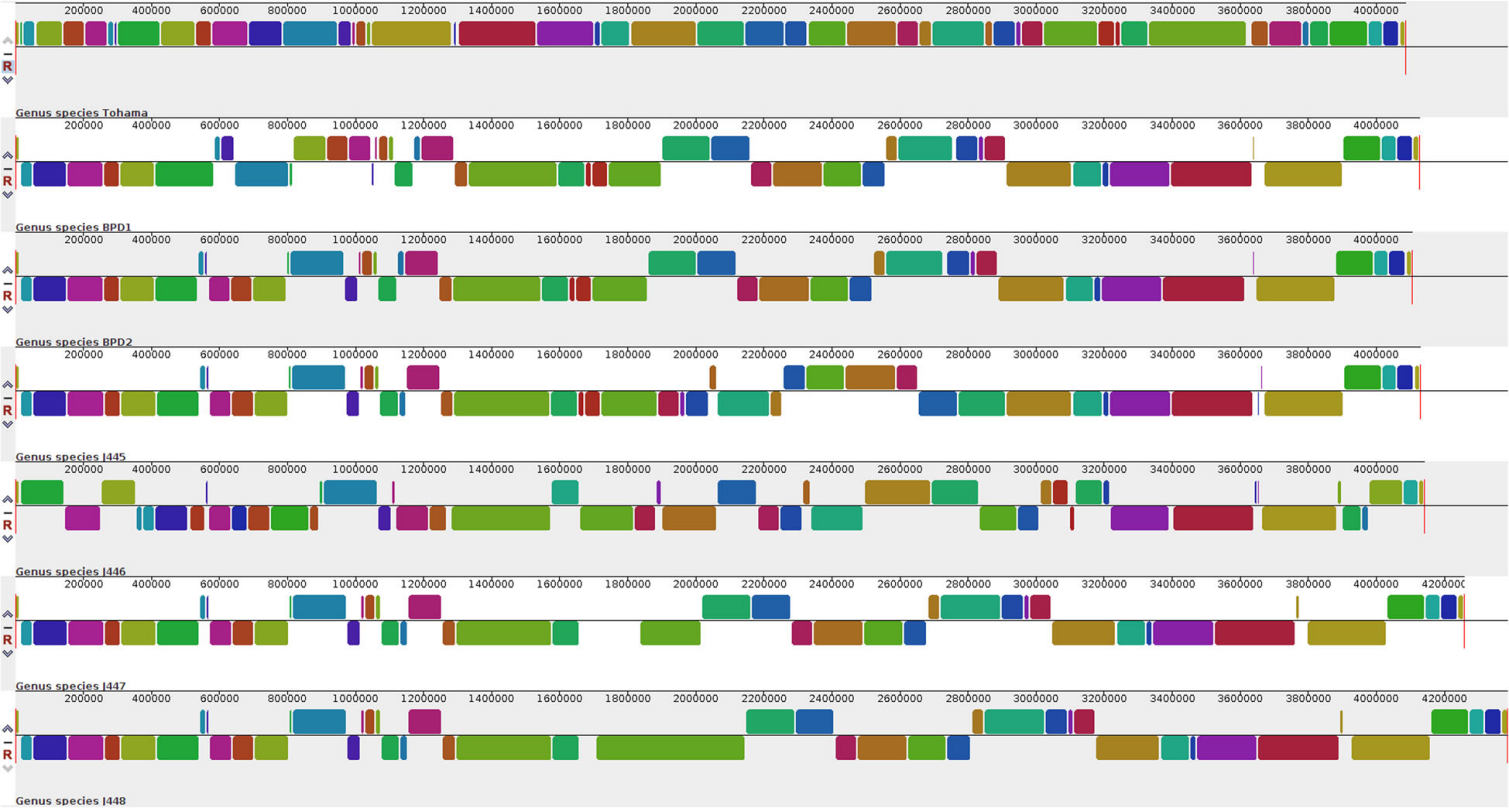
Whole genome alignment using Mauve. Whole-genome alignment of *B. pertussis* vaccine strains (J445, J445, J446, J448) and clinical strains (BPD1, BPD2) against reference strain Tohama-I using a progressive Mauve2.0 algorithm. Colored outlined blocks surround regions of the genome sequence that is conserved with other aligned genomes called a localized collinear block (LCBs). An LCB block below the central black line indicates an inversion event relative to Tohama-I

Genome structure has important effects on bacterial phenotypes and the evolution of bacterial genomes. To detect overall chromosomal rearrangements, deletions, duplications among vaccine strains, and clinical strains with the reference genome, we aligned the genomes using Mauve v2.0 [82]. The multiple whole-genome alignment was conducted using the progressive alignment algorithm implemented in Mauve v2.0 using default parameters. Alignment output generated 5,064,183 root alignment length and 103 super intervals. Mauve showed a total of 56 localized collinear blocks (LCB) in the analyzed genomes, which suggest the presence of rearrangement observed in vaccine and clinical genomes as compared to reference genome (Additional file 7). An LCB represents a conserved region within genomes with exactly matched sequences that are shared by genomes aligned. Some LCBs are too small to display on the figure (Fig. 7). LCB boundaries were found using two programs, project And Strip, and makeBadgerMatrix, to generate LCB boundary files from the Mauve alignment.

Mauve detected a total of 20 inversions observed in clinical isolates BPD1 and BPD2 with reference Tohama-I, where these 20 blocks varied in size (Additional file 7). The major inversion is seen in BPD1 (located at 2142133–2379723) with LCB length 237,590 and in BPD2 (located at 3172139–3420774) with LCB length 248,635 was detected. Few small-scale inversions were also observed (Additional file 7). Few chromosomal arrangements like translocations were also found within BPD1 and BPD2 when compared to reference strain and vaccine strains.

## Discussion

Antigenic divergence among circulating strains of *B. pertussis* is reported globally [69–73]. The impact of WCV on the circulating strains needs to be established. Manufacturing of WCV involves the use of more than one strain. The strains used in manufacturing vary globally. Therefore, genetic characterization of vaccine strains is important. Whole genome based comparative genomics offers high resolution data to study sequence and structural related variations. Till recently, such data was available for vaccine and circulating strains from countries using ACV. We report here comparative genome analysis of vaccine strains with the reference strain, clinical isolates reported from India and isolates from countries using ACV.

The study includes analysis of genome characteristics, genomic structure and rearrangement, mobilome analysis, phylogeny, virulence factor typing, MLST and cgMLST analysis of pertussis strains. Comparative whole genome analysis of clinical isolates reported from India displayed ≥95% genome similarity with reference and WCV strains used in India. Further, mobilome analysis of clinical isolates from India suggests slight variation in number of pseudogenes, ISs among the strains. These differences in pseudogenes could be ascribed to the fact that vaccine strains are stored frozen in optimized conditions and have limited exposure to host. IS elements especially IS481 in *B. pertussis* strains is reported to vary among circulating strains [55–57]. Increased copy numbers of IS481 were also observed in Indian clinical isolates. A higher number of IS elements in monomorphic populations such as *B. pertussis* are known to *provide* competitive advantage because of its role in genome reduction and genomic rearrangements [53, 54]. Bacteriophages contribute actively to bacterial evolution by integrating and excising from the genome [58]. In certain conditions, they provide new genetic properties to the bacterial host and leading to the development of new virulence within species. Indian clinical isolates and WCV strains showed the absence of phages, CRISPR and plasmids amongst the genomes which is significant towards the minimal genome plasticity. IS elements among the strains J445, J446, J447, J448, BP165 and BPD1 did not showed association with any virulence-associated genes and its controlling elements. Overall, mobilome analysis observations are significant towards the stability of vaccine and clinical genomes.

Antibiotic treatment and prophylaxis constitute an essential part of interventions used to treat bacterial diseases. Macrolides including azithromycin, clarithromycin, and erythromycin remain the mainstay of treatment for pertussis [60]. Few reports have demonstrated antibiotic resistance against antibiotics like macrolide, erythromycin (minimum inhibitory concentration MIC > 256 IU/L) in circulating strains of *B. pertussis* [61, 62]. Antibiotic resistance plays a key role in the resurgence of infectious disease. First erythromycin resistance cases were reported from the United States in 1994 [85]. Resistant strains were also reported from countries like France and China [61, 62]. In a recent study, antibiotic resistant strains were reported from Iran which is using WCV in its immunization programme [86]. This led us to monitor the emergence of antibiotic resistance in strains reported from India. Absence of antibiotic resistance genes observations in Indian clinical isolates need confirmatory studies with pan India isolates. Such data will be further useful to decipher how antibiotic resistance evolves in countries which are using WCVs as selection pressure may be different from what we have observed in countries which are using ACV.

Clonal expansion of *B. pertussis* circulating strains is reported in countries using ACV [28]. However, there is scarcity of data available from countries using WCV. Core genome based phylogenetic analysis of WCV strains showed close evolutionary relationship with reference Tohama-I strain and Indian clinical isolates suggesting lesser genetic diversity in Indian strains. Indian clinical isolates as compared to isolates reported from ACV were found to cluster distinctly, indicating minimal intermixing from global strains. Use of WCV in primary immunization programs can be one possible reason for lesser diversity in Indian strains. MLST and genotyping analysis was carried out for WCV strains. Out of 4 strains of WCV, 3 strains were found to have ST-2 profile, where 1 strain showed ST-1 and allele profile of virulence associated genes was similar in vaccine and clinical strains. Thus, a vaccine manufactured using combination of such strains may allow better coverage against circulating strains. However, this needs confirmation with a greater number of isolates from India and other WCV using countries.

Our initial findings do support the emerging hypothesis that *B.pertussis* evolution is associated with vaccine usage and this evolution is moving at different rates in different countries. However, it needs validation using a larger collection of clinical isolates from countries using WCV like India. Nevertheless, this study provides necessary data on WCV strains to study the evolution and diversity of *B.pertussis* in developing countries.

## Conclusions

The study provides a comprehensive genomic analysis of WCV strains and clinical isolates from India which will be useful in facilitating surveillance of pertussis in India and its comparisons with globally reported trends in *B. pertussis* populations.

## Methods

### Strain selection

A total 5 vaccine (J445, J446, J447, J448, BP165), 2 clinical (BPD1, BPD2) and 1 reference strain (Tohama-I) were used in this study. Four strains (J445, J446, J447, and J448) were used in manufacturing of WCVs [42]. Strain J445 and J446 were procured from the culture collection of the Rijks Institute voor de Volksgezondheid, Netherlands. Strain J447 and J448 were obtained from the Lister Institute in London, England. Strain BP165 is a US clinical isolate procured from the Center for Biologics Evaluation and Research (CBER, United States) and used in manufacturing of acellular vaccines [43]. Strain BPD1 and BPD2 were clinical strains reported from India were used. Tohama-I was used as reference genome. Accession number of all the strains is provided in Table 1. Genomic sequences of 166 isolates used in development core genome MLST database of *B. pertussis* strains were used in this study. These isolates represent region correspond to France, US and UK [45]. Of these 166 isolates, 55 isolates from France corresponded to groups of intrafamilial or of multiple isolates from the same patient and randomly selected cocirculating isolates. Out of the remaining 109 isolates, 55 isolates corresponding to 2 outbreaks of pertussis that observed in ACV using countries like US and 54 isolates from UK.

### Genome sequencing and annotation

Genomes of all strains were compared with reference strain Tohama-I. J445, J446, J447, J448 vaccine strains were sequenced using a combination of the PacBio RSII and Illumina MiSeq platforms as described previously [42], and genome sequences were deposited in GenBank databases under accession numbers available in Table 1 [38]. Genomic DNA extraction of fifth vaccine strain BP165 was performed using the Genomic DNA Clean and Concentrator − 10 Kit (ZYMO Research) as per the manufacturer’s instructions, and DNA was quantified using nanodrop and Qubit. Sequencing was performed by preparing shotgun libraries using NEB Next Ultra DNA library prep kit. Sequencing was performed using platform Illumina MiSeq with 150 read data length (100X coverage) using paired-end sequencing. The whole-genome sequence of strain BP165 has been deposited in GenBank under the accession number RSFF00000000 [43]. One hundred and sixty-six isolates available from ACV countries available from databases were used for phylogenetic analysis [45]. Annotation of all the sequenced genomes used in this study (J445, J446, J447, J448, BP165 and BPD1, BPD2) was completed using automated annotation tool, to avoid the possible deviations due to different annotation methods [74].

### Pan-genome analysis

Pan-genome analysis was performed using a bacterial pan-genome analysis (BPGA-ultra-fast bacteria) pipeline [50]. To depict the core and accessory genomes in each genus, a reciprocal best hit search using the BPGA software was performed. Pan-core plot against combinations will give core and pan-genome boxplot, and dot plot was generated using the desired number of unique combinations of genomes. Orthologous groups (OGs) of genes were observed using the orthoMCLv.2.0.9 software package (default parameters-value cutoff of 10–5, per cent match cutoff 50%, MCL algorithm inflation value 1.5). Homologous clusters from OrthoMCL were compiled to show shared and unique genes [75].

### Mobilome analysis

All strains were investigated for the presence of mobile genetic elements (MGEs) like bacteriophage, Insertion sequences, Genomic islands and Prophages. Potential prophage sequences in the genome were found and categorized (intact, incomplete or questionable) using PHASTER tool (http://phaster.ca/). A prediction of whether phage region has an intact or incomplete prophage was based on score value, which is for intact (> 90), questionable (70–90), and for incomplete (< 70) [59]. Insertion sequences located within the genome were identified using ISfinder (https://isfinder.biotoul.fr/) [76]. Additionally, each of the genomes was manually reviewed for the copy number of transposes sequences. Genomic islands (GIs) were predicted by using IslandViever3, including IslandPick, IslandPath-DIMOB, and SIGI-HMM [77]. Pseudogenes were predicted using NCBI Prokaryotic Genome Annotation Pipeline [78].

Virulence genes were identified using Virulence Finder, and Antibiotic resistance genes (ARGs) using a combination of ResFinder and the Comprehensive Antibiotic Resistance Database (CARDs) [79].CRISPER Finder (http://crispr.u-psud.fr/) was used to identify clustered regularly interspaced short palindromic repeats (CRISPRs) [80].

### Whole-genome comparison

A BLAST Atlas comparing all genomes to the reference genome was generated by GView comparison tool [81]. Genome organization was studied by aligning sequenced genomes using a progressive Mauve algorithm (version 2.0) with default parameters [82]. All the tools used with the default settings.

### MLST and cg-MLST analysis

MLST was performed using *www.pubmlst.org**(PubMLST) Bordetella spp.* database based on housekeeping genes (adenylate kinase, fumarate hydratase class II, aromatic amino-acid aminotransferase, isocitrate dehydrogenase, cytosol aminopeptidase, and phosphoglucomutase) and genotyping of antigenic determinant genes (pertussis toxin subunit 1, pertactin, filamentous hemagglutinin, fimbriae2, fimbriae3, outer-membrane protein Q, *Bordetella* anti-transporter-protein C, adenylate cyclase toxin, virulence-activated gene 8, and tracheal colonizing factor) was also studied. The allele of each gene and the sequence type (ST) were defined according to the public database [33, 46]. cgMLST was analyzed using a publicly available web-accessible genotyping platform (https://bigsdb.pasteur.fr/bordetella) [45]. Briefly, genome assemblies were compared using BLASTN to the reference alleles of 2038 predefined gene loci, as previously described [51]. We evaluated individual comparison of each strain allelic profiles and recorded the number of mismatches, and defined cgST profile for each isolate according to cgMLST scheme of 2038 core gene loci. To derive a phylogenetic tree based on cgMLST loci, we extracted and aligned the amino acid sequences of all loci with MAFFTv7, as previously described [83]. We used IQ-TREEv1.5.4 to infer a maximum likelihood phylogenetic tree based on alignment of the sequences from each 2038 cgMLST loci. We assessed branch support with bootstrap (1000 replicates). Phylogenetic tree annotation and visualization were performed using iTOL [78, 84]. Duplicates and lost genes were predicted solely based on Rapid Annotation using Subsystem Technology (RAST) annotation pipeline. The complete list of genes annotated in all vaccine and clinical genomes were evaluated and compared with reference genome [87].

## Supplementary information

**Additional file 1.** Genome neighbor report analysis of vaccine (J445, J446, J447, J448) and Clinical (BPD1, BPD2) strains. NCBI genome neighbor report analysis of vaccine and clinical strains suggesting % symmetric identity with total available genomes in NCBI databases.

**Additional file 2. **Mobile genetic elements (Insertion Sequences, Prophages) identified in the Bordetella vaccine and clinical strains. Insertion sequences identified in eight *B. pertussis* vaccine strains (J445, J4445, J446, J448, BP165) and clinical strains (BPD1, BPD2) and reference strain Tohama-I using IS Finder with describing details about blast matched values, e values and IS families. The second sheet listing phage regions identified in all strains. Details of phage region with its nature and sequence are described in the last sheet.

**Additional file 3. **Genomic island region observed for *B. pertussis* vaccine strains. List and details of all GI regions observed with coded genes information for strains (J445, J4445, J446, J448, and BP165) and clinical strains (BPD1, BPD2). Table describing the list of genes observed in each genomic island with a total length of island region (bp), start and end region, a gene present in particular island, Gene start and gene end, and product expressed by a particular gene.

**Additional file 4.** cgMLST Analysis. Table describing allelic profile observed for total 2038 genes with locus of each gene, allele number, and length of the gene, contigs number and starts, and end position of each gene observed in respective strain.

**Additional file 5.** List of genes absent and duplicated in strains. List of duplicated genes observed in the vaccine (J445, J4445, J446, J448, and BP165) and clinical strains (BPD1, BPD2) as compared to the reference strain. Table describing genes duplicated in particular strain (Highlighted part) with contig number, start and end position of genes, total length, and gene product with subsystem annotation.

**Additional file 6.** BLAST ATLAS. List of genes identified during BLAST ATLAS genome comparison with reference genome Tohama-I.

**Additional file 7.** Permutation matrix file. Permutation matrix file generated from MAUVE progressive alignment containing information about total LCB blocks observed during alignment generated and their relative position in each genome.

## Data Availability

The datasets generated or analyzed during the current study are available in the National Center for Biology Information (NCBI), GenBank repository, [under accession numbers as CP017402, CP017403, CP017404, CP017405, RSF00000000, CP034182, CP0134101]”. Most other datasets obtained from web-based sources are included as weblink in “Method” section and “Additional files”.

## Abbreviations

WCVs: Whole-cell vaccines
ACVs: Acellular vaccines
cg-MLST: core genome multilocus typing
MLST: Multi-locus sequence typing
COG: Cluster of orthologous genes
MGEs: mobile genetic elements
ISs: Insertion sequences
MGEs: Mobile genetic elements
ST: Sequence type
GIs: Genomic islands
ptx: Pertussis toxin
prn: Pertactin
fim: Fimbrae
FHA: Filamnetous hemaglutinin
tcf: Tracheal colonization factor
CDS: Coding sequences
NCBI: National centre for Biological Sciences
CRISPER: Clustered Regularly Interspaced Short Palindromic Repeats
HGT: Horizontal gnee transfer
BapC: Bordetella associated protein c
cyaA: Adenylate cyclase
vag8: Virulence associated gene 8
LCB: Local collinearized Bloack
PATRIC: Pathosystems Resource Integration Center
CC: Clonal complex
iTol: Interactive Tree of Life
RAST: Rapid Annotation using Subsystem Technology.
